# Podocyte Infolding Glomerulopathy: A Case Series Report and Literature Review

**DOI:** 10.3390/jcm12031088

**Published:** 2023-01-30

**Authors:** Yunlin Feng, Wei Wang, Yurong Zou, Tingyu Chen, Wei Wang, Guisen Li, Amanda Y. Wang, Ping Zhang

**Affiliations:** 1 Department of Nephrology, Sichuan Provincial People’s Hospital, University of Electronic Science and Technology of China, Chengdu 610072, China; 2 Chinese Academy of Sciences Sichuan Translational Medicine Research Hospital, Chengdu 610072, China; 3 The Renal and Metabolic Division, George Institute for Global Health, University of New South Wales, Sydney 2042, Australia; 4 Nephrology Department, Chengdu First People’s Hospital, Chengdu 610021, China; 5 Concord Clinical School, University of Sydney, Sydney 2006, Australia; 6 The Faculty of Medicine and Health Sciences, Macquarie University, Sydney 2109, Australia

**Keywords:** podocyte infolding, glomerulopathy, case series, *INF2*, literature review

## Abstract

Background: Podocyte infolding glomerulopathy (PIG) is a peculiar and very rare manifestation in renal pathology. Its underlying pathogenesis mechanism and clinical characteristics remain unclear due to sparse reports. Objective: To further elucidate the clinical profile of PIG by carefully reporting our four cases and a comprehensive review of cases in the literature. Methods: This study retrospectively reviewed four cases of PIG from 2010 to 2022 in our centre. Clinical and pathological profiles were reported. PIG cases in the literature were searched in the MEDLINE database and analysed together with our cases. Results: Four cases of PIG identified from our centre and 40 cases from the current literature were reported. The pooled analysis of these 44 cases indicated 79.5% (35/44) were females, 93.2% (41/44) were East Asians, and 63.6% (28/44) were reported in Japan. The average age was 42.0 ± 12.5 years old. The average amount of proteinuria at the time of renal biopsy was 3.06 ± 3.2 g/day. The most reported comorbidities were connective tissue diseases, mainly systemic lupus erythematosus, and 20.5% (9/44) of the cases did not have any contaminant disease. Most of the cases (81.8%, 36/44) had been treated with immunosuppressants, of which a combination of corticosteroids and one other type of immunosuppressant was most commonly reported. In addition, 45.4% (20/44) and 34.1% (15/44) of the cases had achieved complete response and partial response, respectively, after treatment. Whole exosome sequencing indicated mutations in the *INF2* gene. Conclusions: PIG is a rare condition and seen in relatively younger populations, often associated with connective tissue diseases clinically and one or two other glomerulopathies histologically. The outcomes following immunosuppressive treatment are relatively good. Mutations in *INF2* might be involved in the development of PIG; however, the implications of these results need to be investigated.

## 1. Introduction

Podocyte infolding glomerulopathy (PIG) is a very rare phenomenon that was first reported as a unique pathological manifestation in 1992 [1] in Japan and officially proposed as a new disease entity in 2008 by the Japanese Society of Nephrology in 2008 [2]. PIG is characterized by peculiar microstructures of microspheres, microtubules, or both within the thickened glomerular basement membrane (GBM), accompanied by podocyte infolding [2]. It is not included as a separate category in the current classification of glomerular diseases. Thus far, most reported cases have been in East Asians.

The underlying pathogenesis mechanism of PIG remains unclear. Successive pathology sections analysis indicated that PIG might be a continuous process of podocyte processes from primary infolding to bubbling [2]. The initiation factor of the primary infolding is not clear. The only gene reported to be involved in PIG so far is SMARCAL1, detected by whole exosome sequencing in a child with Schimke immune-osseous dysplasia (SIOD) [3]. However, the contributions of these mutations to the development of PIG were unclear.

The clinical features are highly diversified in PIG. It has been reported that PIG is associated with other diseases, mostly connective tissue diseases, including systemic lupus erythematosus (SLE) and Sjogren Syndrome (SS) [4,5,6,7]. However, there are also cases of PIG that were not associated with any disease entity [2,8,9]. Whether PIG is a new disease entity or just a specific renal pathological finding of coexisting diagnoses remain controversial.

To improve our understating of PIG, we reported here the largest PIG case series study from a single center thus far and comprehensively reviewed the reported cases in the literature alongside our case series.

## 2. Case Presentation

### 2.1. Case 1

**Figure 1:**
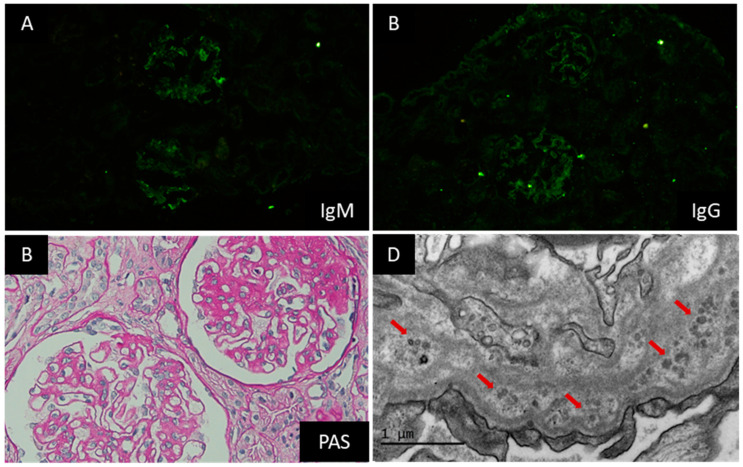
Representative pathological images of renal biopsy specimen form Case 1. Note: IF staining was positive for IgM (**A**) and IgG (**B**) in capillary walls and mesangial regions. Light microscopy indicated mild mesangial matrix expansion and diffuse thickening of GBM ((**C**), PASM + Masson, 400×). Electron microscopy showed thickening of the GBM, diffuse podocyte foot process effacement, and prominent microspheres forming clusters (red arrow) within the GBM ((**D**), 20,000×).

This patient was a 26-year-old lady, without a known history, who presented with intermittent edema in both lower extremities for one month and denied any accompanying symptoms. Physical examination on admission indicated no remarkable findings except for mild pitting edema in both legs. Remarkable laboratory investigations at this admission were as follows: WBC 12.60 × 10^9^/L, HGB 123 g/L, PLT 161 × 10^9^/L, proteinuria 2.74 g/day, hematuria (−), serum creatinine (Cr) 56 μmol/L, complement C3 0.96 g/L, and total cholesterol (TC) 5.14 mmol/L. All autoimmune antibodies tested negative. A renal biopsy was done on the third day after admission. Immunofluorescence (IF) staining was positive for IgG and IgM in the capillary walls and mesangial regions (Figure 1A,B) and negative for subtypes of IgG and PLA2R antibodies. Light microscopy found global glomerulosclerosis in 2/15 glomeruli, segmental sclerosis with synechial attachment to Bowman’s capsule in 7/15 glomeruli, mild mesangial matrix expansion, and diffuse thickening of glomerular basement membrane (GBM) (Figure 1C). Marked vacuolar degeneration and granular degeneration in renal tubular epithelial cells and mild interstitial fibrosis and tubular atrophy (<5%) were also noticed. Electron microscopy found thickening of the GBM, diffuse podocyte foot process effacement, and prominent microspheres forming clusters within the GBM (Figure 1D). She was diagnosed with focal segmental glomerular sclerosis (FSGS), membranous nephropathy, and PIG. Corticosteroid therapy was prescribed, starting with 50 mg of prednisone daily. Her proteinuria began to decrease after 2 months and remained around 1 g/day. The prednisone was tapered gradually from the third month and stopped at the sixth month. Eighty milligrams of Valsartan daily was prescribed afterward. Her proteinuria remained in the range of 0.7–1.2 g/day, and her serum Cr remained normal.

### 2.2. Case 2

**Figure 2:**
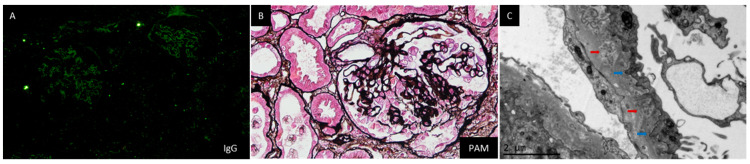
Representative pathological images of renal biopsy specimen form Case 2. Note: IF staining was positive for IgG (**A**) in capillary walls. Light microscopy showed segmental sclerosis with podocyte proliferation in 3/14 glomeruli and diffuse thickening of GBM ((**B**), PASM + Masson, 400×). Electron microscopy showed diffuse podocyte foot process effacement, multiple podocyte infolding (blue arrow), and prominent microspheres forming clusters (red arrow) within the GBM ((**C**), 20,000×).

This patient was a 47-year-old gentleman, without a known history, who presented with edema in both lower extremities for more than 10 days. He complained of progressive edema starting from both ankles to his knees. Foamy urine was also noticed. At admission, physical examination only found pitting edema in both legs. Remarkable lab investigations at this admission were as follows: WBC 9.25 × 10^9^/L, HGB 163 g/L, PLT 280 × 10^9^/L, proteinuria 9.96 g/day, hematuria (−), urine albumin-to-creatinine 5277.5 μg/mg, Cr 94.4 μmol/L, C3 0.95 g/L, and TC 6.01 mmol/L. All serum autoimmune antibodies tested negative. The result of the anti-PLA2R antibody was also negative. A renal biopsy was done on the third day after admission. IF staining was positive for IgG in the capillary walls (Figure 2A) and negative for IgM, IgA, subtypes of IgG, and PLA2R. Light microscopy found segmental sclerosis with podocyte dysplasia in 3/14 glomeruli, marked hyperplasia of overlying epithelial cells in 2/14 glomeruli, and diffuse thickening of GBM (Figure 2B). Mild interstitial fibrosis and tubular atrophy (<5%) was also noticed. Electron microscopy found diffuse podocyte foot process effacement, multiple podocyte infolding, and prominent microspheres forming clusters within the GBM (Figure 2C). The diagnosis was FSGS and PIG. His treatment included 30 mg of prednisone daily and 1 mg of tacrolimus twice daily. The proteinuria began to decrease after two months and then remained in the range of 1.2–1.9 g/day. The prednisone was tapered gradually from the third month and stopped at the end of the seventh month. Tacrolimus still continued at the time of this report. His serum Cr was normal throughout the follow-up period.

### 2.3. Case 3

**Figure 3:**
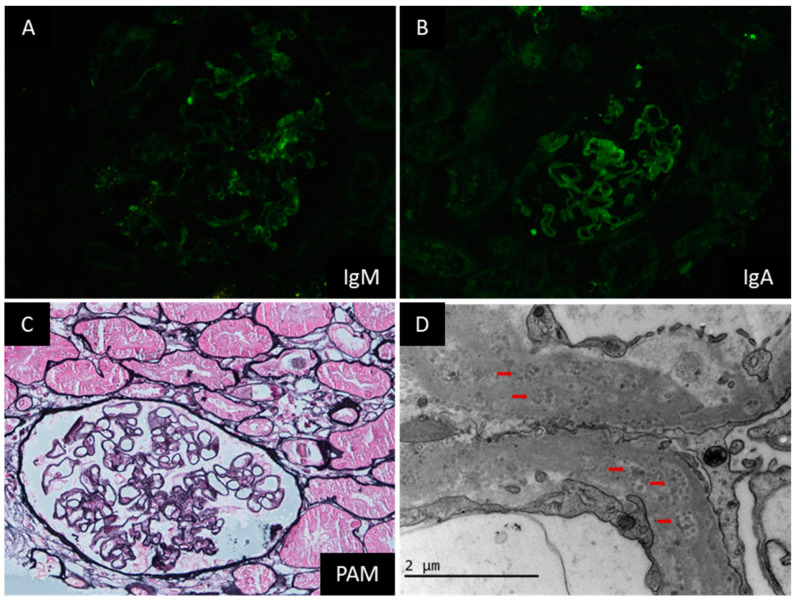
Representative pathological images of renal biopsy specimen form Case 3. Note: IF staining was positive for IgM and IgA in mesangial regions (**A**,**B**). Light microscopy indicated segmental sclerosis with synechial attachment to Bowman’s capsule in 2/20 glomeruli, mild mesangial matrix expansion, and diffuse thickening of GBM ((**C**), PASM + Masson, 400×). Electron microscopy found diffuse podocyte foot process effacement and prominent microspheres (red arrow) in the GBM ((**D**), 20,000×).

This patient was a 48-year-old lady with a history of SLE for 20 years who presented with edema in both lower extremities for one week. After the diagnosis of SLE, she had been initially treated with prednisone and mycophenolate mofetil (MMF) for five years, which was then changed to prednisone and tacrolimus. She claimed having a renal biopsy 10 years ago, but the detailed results were unavailable. At this admission, she was receiving 10 mg of prednisone daily, 1.0 mg of tacrolimus twice daily, 100 mg of hydroxychloroquine, and 80 mg of valsartan daily. Physical examination indicated mild edema in both lower extremities and old-standing butterfly erythematosus on both cheeks. Remarkable laboratory investigations at this admission were as follows: WBC 2.31 × 10^9^/L, HGB 10^5^ g/L, PLT 119 × 10^9^/L, proteinuria 4.37 g/day, hematuria (−), urine albumin-to-creatinine 5967.6 μg/mg, Cr 59.3 μmol/L, C3 0.727 g/L, ANA 1:100 (+), anti-SSA-Ab (+++), and anti-Ro52-Ab (+++). A repeat renal biopsy was done on the fourth day after admission. IF staining was positive for IgA and IgM in the mesangial regions (Figure 3A,B) and PLA2R in the capillary walls and negative for IgG and subclass IgG. Light microscopy found global glomerulosclerosis in 6/20 glomeruli, segmental sclerosis with synechial attachment to Bowman’s capsule in 2/20 glomeruli, mild mesangial matrix expansion, and diffuse thickening of GBM (Figure 3C). Focal interstitial fibrosis and tubular atrophy (about 15%) was also noticed. Electron microscopy found diffuse podocyte foot process effacement and prominent microspheres within the GBM (Figure 3D). The diagnosis was membranous nephropathy and PIG. The patient was then treated with 360 mg of belimumab once a month based on her existing combination therapy of prednisone and tacrolimus. Her proteinuria began to decrease from 5.43 g/day at discharge to 0.91 g/day after one month and remained in the range of 0.9–1.4 g/day for eight months, but it was gradually increased to 2.0–3.8 g/day afterwards. The triple regimen continued at this report. Her serum Cr was normal throughout the follow-up period.

### 2.4. Case 4

**Figure 4:**
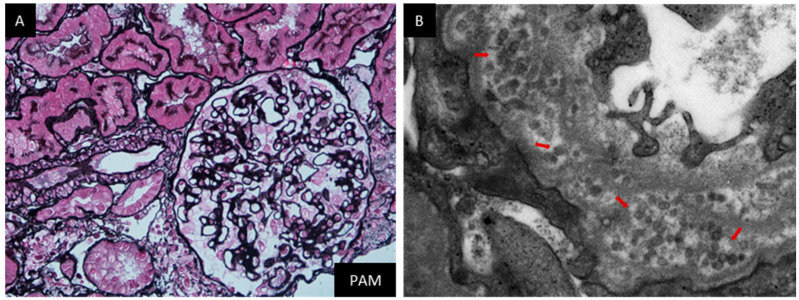
Representative pathological images of renal biopsy specimen form Case 4. Note: Light microscopy showed mild mesangial matrix expansion and mild thickening of GBM ((**A**), PASM + Masson, 400×). Electron microscopy showed diffuse podocyte foot process effacement, mild podocyte infolding, and prominent microspheres (red arrow) within the GBM ((**B**), 50,000×).

This patient was a 57-year-old lady, without a known history, who presented with intermittent edema in both lower extremities for more than two months. She denied any other symptoms. Physical examination at admission indicated mild edema in both lower extremities. Remarkable lab investigations at this admission were as follows: WBC 5.09 × 10^9^/L, HGB 137 g/L, PLT 164 × 10^9^/L, proteinuria 3.54 g/day, hematuria (−), serum Cr 33.3 μmol/L, serum ALB 26.8 g/L, C3 0.91 g/L, ANA 1:100 (+), anti-SSA-Ab (+), and anti-AHA-Ab (+). A renal biopsy was done on the third day after the admission. IF staining was nonspecific for IgG, IgA, IgM, C3, C1q, and all subtypes of IgG and PLA2R. Light microscopy found mild mesangial matrix expansion and mild thickening of GBM, supporting mild glomerular abnormality (Figure 4A). Scattered interstitial fibrosis and tubular atrophy (about 5%) was also noticed. Electron microscopy showed diffuse podocyte foot process effacement, mild podocyte infolding, and prominent microspheres within the GBM (Figure 4B). The diagnosis was mild mesangial proliferative glomerulopathy and PIG. The patient was initially treated with 45 mg of prednisone daily. Her proteinuria began to decrease from 4.2 g/day at discharge to 0.48 g/day after two months. The prednisone was tapered gradually from the third month and stopped at the eighth month. Her proteinuria increased again to 4 g/day after one year. The patient was then treated with 10 mg of prednisone daily and 1.0 mg of tacrolimus twice daily to date. Her proteinuria was maintained in the range of 2.2–3.2 g/day afterwards. Her serum Cr was normal throughout the follow-up period.

### 2.5. Whole Exosome Sequencing Results

**Figure 5:**
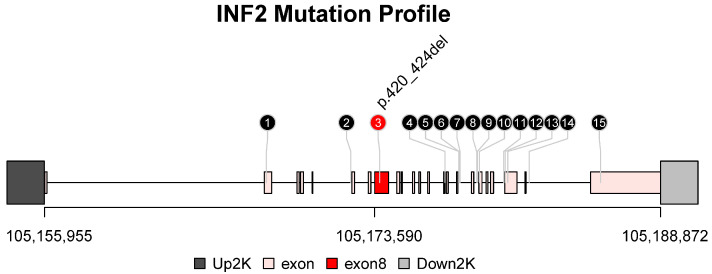
Lolliplot illustrating the mutation profile in *INF2* based on the whole exosome sequencing results of the patients. Note: All SNPs and INDELs in *INF2* were plotted. The numbers inside the circle represented the mutation order. The start and end positions were indicated on the horizontal axis. All exons were filled in mistyrose. The upstream and downstream 2K regions were coloured with dark grey and light grey, respectively. Harmful mutations were represented with red circles, while harmless mutations were represented with black circles.

Whole exosome sequencing of three of these four patients indicated mutations in the *INF2* gene. All single nucleotide polymorphisms (SNPs) and insertion–deletion mutations (INDELs) were filtered to retain only variants classified as “PASS”. The retained variants were loaded into the R environment (v4.2.0; R Foundation for Statistical Computing, Vienna, Austria) and then visualized using the lolliplot function, described previously [10], using a Bioconductor package trackViewer (version 1.32.1; Bioconductor, Boston, MA, USA) (Figure 5). Among all SNPs and INDELs, the third mutation p.420_424del, a non-frameshift deletion located in exon8, was a harmful mutation and labelled with a red circle. The underlying meaning of these findings were to be investigated due to the small sample size of this study.

### 2.6. Literature Review

A comprehensive literature review on PIG identified 40 cases (see Section S1 in Supplementary Materials). All of them were included in the pool analysis alongside the four cases being reported from our center. The detailed characteristics of clinical and histological profiles of these 44 cases are shown in Table 1 and Table 2, respectively.

Among these 44 cases, 79.5% (35/44) were females, 93.2% (41/44) were East Asians, and 63.6% (28/44) were reported in Japan (see Figure S1). The average age was 42.0 ± 12.5 years old. At the time of renal biopsy, the median (interquartile range) serum creatinine level was 61.9 (52.3) μmol/L, and the average amount of proteinuria was 3.06 ± 3.2 g/day.

An analysis of clinical profiles of these cases indicated the most reported comorbidities were connective tissue diseases, mainly systemic lupus erythematosus (see Figure S2), and 20.5% (9/44) of the cases did not have any contaminant disease. Hematuria was not common. Most of the cases (81.8%, 36/44) were treated with immunosuppressants, of which a combination of corticosteroids and one other type of immunosuppressant was most reported. Furthermore, 45.4% (20/44) and 34.1% (15/44) of the cases have achieved complete response and partial response, respectively, after treatment.

An analysis of the histological profiles of renal biopsies indicated 31.8% (14/44) of the cases were all negative for immunofluorescence (IF) staining (see Figure S3). The most seen deposit on IF staining was IgG. Dense deposits on electron microscopy were not common and only seen in 27.2% (12/44) of the cases.

## 3. Discussion

Here we reported the largest case series of PIG from a single center so far. The pooled analysis of our cases and the cases in the literature indicated PIG was seen in relatively young populations, often associated with connective tissue diseases clinically and one or two other glomerulopathies histologically. The outcomes following immunosuppressive treatment were relatively good. We also reported for the first time that mutations in *INF2* might be involved in the development of PIG; however, the significance of these results were yet unclear.

PIG is a peculiar and very rare pathological manifestation which lacks commonality both clinically and histologically. Although it was proposed as a disease entity in 2008 [2], PIG shows great heterogeneities in multiple aspects. It has been reported in both primary and secondary glomerular diseases and has been observed without any marked glomerular nephropathy. As seen in our literature review, SLE is the most reported comorbidity (17/44, 38.6%) [2,4]. Other connective tissue diseases, such as primary biliary cirrhosis, rheumatoid arthritis, mixed connective tissue disease, and Sjogren syndrome, were also reported [5,6,8,11]. Viral infection was reported in a few cases [2,4]. Membranous nephropathy (MN) and focal segmental glomerular sclerosis (FSGS) were the most common histopathological findings associated with PIG, with 12 and 8 cases reported in the literature and this case series, respectively. There were also a significant proportion of cases that showed only mild glomerular changes. In case three, electron-dense deposits were observed in the thickened GBM, which could be explained by her SLE. This should be differentiated from thickened GBM seen in diabetic nephropathy, smoking and obesity, and when uniform thickening of GBM can also be seen but could be distinguished based on the lack of electron-dense deposits and organized ultrastructure. Thickening of GBM in PIG might be caused by a loss of balance between degradation and biosynthesis of the podocyte matrix [2].

The role of PIG in the pathogenesis of glomerulopathies is still unclear. There are a few hypotheses. For example, it was reported that the formation of PIG might be related to the role of special types of complement activation in situ on the microstructures [18]. Since this phenomenon is a dynamic process from primary infolding to bubbling of podocyte foot processes, which has been observed in serial section electronic microscopy, we also suspected that deficits of cytoskeletal structures might be responsible for this change. The whole exosome sequencing results indicated three of the four cases in our case series carried mutations in *INF2*, which is a known causative gene of genetic FSGS [19]. *INF2* is extensively expressed in renal podocytes, of which the mutations cause the mislocalization of *INF2* in podocytes and impaired actin dynamics, subsequently damaging the integrity of the glomerular filtration barrier [20,21,22]. Although our whole exosome sequencing results indicated all four patients carried mutations of *INF2*, these findings did not support the specifically causative relationship between *INF2* mutations and PIG. Future work on potential changes in cytoskeletal structures of podocytes in PIG might help to shed light on this link and will be our next step.

Clinical outcomes of PIG were relatively good. In this review, most cases (34/44, 77.3%) received immunosuppressive treatment consisting of corticosteroids only or with a combination of other immunosuppressants, and nearly 80% (35/44) of the patients had achieved at least partial response. Only less than 5% (2/44) of the patients had shown progression. The coexistence of connective tissue diseases and PIG were the main indications for immunosuppressive treatment. Some patients also received only supportive treatment and had achieved complete or partial response. In our study population, all four patients achieved at least partial response. From this point of view, PIG is relatively benign, even if it is classified as a new disease entity.

To our acknowledge, this is the largest single center case series so far on PIG. PIG is quite heterogenous, both clinically and pathologically. Whether this should be considered as a distinctive disease or merely a pathological representation is inconclusive, according to current understanding. We reported for the first time that mutations in *INF2* might be involved in the development of PIG. However, our study also had some limitations. Our genetic sequencing analysis did not include pedigree studies due to travel restrictions caused by COVID-19. In addition, the mutations in *INF2* lacked specificity for PIG, preventing us from performing any causative analysis. The clinical significance and underlying mechanisms of PIG need further investigations.

## 4. Conclusions

PIG is a peculiar and very rare manifestation in renal histopathology. The pathogenesis and clinical significance of PIG are still unclear. This pooled analysis indicated PIG was seen in relatively younger populations, often associated with connective tissue diseases clinically and one or two other glomerulopathies histologically. The outcomes following immunosuppressive treatment were relatively good. Mutations in *INF2* might be involved in the development of PIG; however, the implications of these mutations need to be investigated.

## Figures and Tables

**Table 1:** Characteristics of clinical profiles of PIG cases reported in the literature and in this case series.

Case No	Sex	Race	Country	Age	Comorbidity	Lab Investigations at Renal Biopsy			Treatment	
						Cr (μmol/L)	Proteinuria *	Hematuria	Therapy	Response
1 [3]	M	Asian	Japan	31	SLE	168.0	0.5 g/day	Absent	PSL (20 mg Qd)	CR
2 [3]	F	Asian	Japan	37	SLE	106.1	1 g/day	Absent	PSL (20 mg Qd), MMF	CR after 6 m
3 [3]	F	Asian	Japan	40	SLE	44.2	1.5 g/day	NR	PSL	NR
4 [3]	F	Asian	Japan	30	SLE	44.2	1.6 g/day	Mild	PSL (pulsex3)	CR after 1 m
5 [3]	F	Asian	Japan	61	SLE, Takayasu’s arteritis	79.6	1.7 g/day	Absent	PSL (40 mg Qd), CsA	PR after 7 y
6 [3]	F	Asian	Japan	29	SLE, hydronephrosis due to lupus cystitis	61.9	1.6 g/day	Absent	PSL (20 mg Qd)	PR after 5 y
7 [2]	F	Asian	Japan	46	SLE, hydronephrosis due to lupus cystitis	44.2	0.6 g/day	Absent	PSL (15 mg Qd)	PR after 2 y
8 [2]	F	Asian	Japan	27	SLE	35.4	2.7 g/day	Absent	PSL (30 mg Qd), MMF	CR after 6 m
9 [2]	M	Asian	Japan	53	SLE, bilateral urethral stone	79.6	3.1 g/day	Present	PSL (30 mg Qd)	CR after 8 m
10 [2]	F	Asian	Japan	23	SLE	44.2	1.8 g/day	Present	PSL (40 mg Qd)	CR after 5 y
11 [2]	F	Asian	Japan	31	SLE	79.6	0.5 g/day	Absent	PSL (20 mg Qd)	CR
12 [2]	F	Asian	Japan	24	SLE, SS	53.0	6.0 g/day	Absent	PSL (pulsex3)	CR after 9 w
13 [2]	M	Asian	Japan	49	PBC, SS, cystitis, finally SLE	97.2	2.2 g/day	Absent	ARB, PSL (40 mg Qd)	CR after 9 y
14 [2]	F	Asian	Japan	20	SLE?	123.8	1.4 g/day	Absent	PSL (40 mg Qd)	CR
15 [2]	F	Asian	Japan	47	RA, pSS	53.0	1.3 g/day	Absent	ARB, PSL (20 mg Qd)	PR
16 [2]	F	Asian	Japan	51	pSS	53.0	3.7 g/day	Absent	PSL (40 mg Qd)	CR after 1 m
17 [2]	F	Asian	Japan	30	MCTD	79.6	0.3 g/day	Absent	PSL (15 mg Qd)	NR
18 [2]	F	Asian	Japan	54	Basedow’s disease	221.0	6.0 g/day	Absent	-	PR after 3 m
19 [2]	F	Asian	Japan	57	Hypothyroidism, chronic thyroiditis	53.0	0.3 g/day	Absent	-	CR after 2 m
20 [2]	M	Asian	Japan	45	Absent	61.9	2.6 g/day	Absent	PSL (50 mg Qd)	PR after 2 m
21 [2]	F	Asian	Japan	42	Ovarian mature teratoma	68.1	7.5 g/day	Present	ARB	PR after 1 m
22 [2]	F	Asian	Japan	69	Absent	79.6	1.6 g/day	Absent	-	PR after 1 m
23 [2]	M	Asian	Japan	46	HBV infection	106.1	4.0 g/day	Absent	Diuretics	PR
24 [2]	M	Asian	Japan	59	Tumor lysis syndrome	450.8	0.6 g/day	Absent	PSL (pulsex3)	NR
25 [2]	F	Asian	Japan	45	Absent	70.7	1.5 g/day	Absent	PSL (30 mg Qd)	CR after 1 y
26 [3]	M	Asian	China	4	SIOD	Normal	3.7 g/day	NR	P, tacrolimus, ACEI	Normal renal function
27 [4]	F	Asian	China	61	SLE, benign ovarian tumor, EBV infection	75.1	2.1 g/day	Present	HCQ, ARB, PSL (40 mg Qd), CTX	PR
28 [5]	F	Asian	India	45	UCTD	145.9	5.8 g/day	Present	High dose PSL, MMF, RTX	PR
29 [6]	F	Asian	China	33	UCTD	36.2	2.1 g/day	Absent	NR	NR
30 [7]	F	Asian	China	27	pSS	168.2	0.6 g/day	Absent	PSL (48 mg Qd), HCQ	Progression
31 [7]	F	Asian	China	23	SLE	47.1	16.8 g/day	Present	PSL (40 mg Qd), HCQ	PR
32 [8]	F	Asian	Japan	14	Absent	48.6	2.35 g/day	Absent	PSL (40 mg Qd)	CR after 9 m
33 [9]	F	Asian	South Korea	44	Absent	39.8	PCR = 67.1/88.29 mg/mg	NR	PSL (10 mg Qd)	CR after 3 m
34 [11]	F	Asian	Canada	52	Prior HBV infection with immunity	61	uACR = 1600 mg/mol	Present	ARB, P (1 mg/kg, Qd), spironolactone	PR, worsened after stopping P
35 [12]	F	Asian	China	52	pSS, Hashimoto’s thyroiditis	NR	NR	NR	NR	NR
36 [13]	M	Asian	Japan	79	MM	113.2	1.4 g/day	Absent	PSL (20 mg Qd)	PR, but death after 2 m
37 [14]	F	Latin American Caucasian	Argentina	38	SLE	Normal	NS range proteinuria	NR	CO + CTX	Good response, but relapsed
38 [15]	F	Asian	Japan	35	Scleroderma	NR	NR	NR	NR	NR
39 [16]	F	NR	USA	60	Absent	61.9	7.3 g/day	Absent	RTX	PR
40 [17]	F	Caucasian	Germany	56	RA	386.3	uACR = 6200 mg/g	Absent	High hose P + RTX	CR
41	F	Asian	China	26	Absent	82.3	2.74 g/day	Present	Prednisone (50 mg Qd, tapered to stop)	PR
42	M	Asian	China	47	Absent	94.4	9.96 g/day	Absent	Prednisone (30 mg Qd) + tacrolimus (1 mg Bid)	PR
43	F	Asian	China	48	SLE	59.3	5.4 g/day	Absent	Prednisone (30 mg Qd) + tacrolimus (1 mg Bid) ± Belimumab (10 mg/kg/w)	PR
44	F	Asian	China	57	Absent	33.3	3.5 g/day	Mild	Prednisone (45 mg Qd)	CR, but relapsed

Abbreviations: ACEI, angiotensin converting enzyme inhibitor; ARB, angiotensin receptor blocker; Bid, twice daily; CO, corticosteroid; Cr, creatinine; CR, complete response; CsA, cyclosporin; F, female; HBV, hepatitis B virus; HCQ, hydroxychloroquine; M, male; m, month; MCTD, mixed connective tissue disease; MM, multiple myeloma; MMF, mycophenolate mofetil; NR, not reported; NS, nephrotic syndrome; P, prednisone; PBC, primary biliary cirrhosis; PR, partial response; PSL, prednisolone; pSS, primary Sjogren syndrome; Qd, once daily; RA, rheumatoid arthritis; RTX, rituximab; SIOD, Schimke immune-osseous dysplasia; SLE, systemic lupus erythematosus; SS, Sjogren syndrome; UCTD, undifferentiated connective tissue disease; w, week; y, year. * The value was the amount of proteinuria per day, unless specified.

**Table 2:** Characteristics of pathological profiles of PIG cases reported in the literature and in this case series.

Case No	Sex	Age	Renal Pathology								
			IF Staining	Hypercellularity	Mesangial Deposit	GBM Thickening	FPE	Microspheres	Microtubules	Dense Deposit	LM Manifestations
1 [2]	M	31	All negative	Absent	Mild	Present	Present	Present	Absent	Absent	MGA
2 [2]	F	37	G, A, C3, C1q	Absent	Mild	Present	Present	Present	Present	Absent	LN Class II
3 [2]	F	40	G, A, C3, C1q	NR	Mild	Present	Present	Present	Present	Absent	LN Class II
4 [2]	F	30	G, A, C3, C1q, C5b-9	Present	Mild	Present	Present	Present	Absent	Mesangium, subendothelial	LN Class II
5 [2]	F	61	G, M, C1q	Absent	Mild	Present	Present	Present	Absent	Present	LN Class II
6 [2]	F	29	All negative	Absent	Absent	Present	Present	Present	Present	Absent	MN
7 [2]	F	46	All negative	Absent	Absent	Present	Present	Present	Present	Absent	MN
8 [2]	F	27	G, A, M, C3, C1q, C5b-9	Absent	Absent	Present	Present	Present	Present	GBM, subendothelial, subepithelial	LN Class V
9 [2]	M	53	All negative	Absent	Absent	Present	Present	Present	Present	Absent	MN
10 [2]	F	23	G	Present	Mild	Present	Present	Present	Present	GBM, subendothelial, subepithelial	LN Class V
11 [2]	F	31	G	Absent	Absent	Present	Present	Present	Present	GBM	LN Class V
12 [2]	F	24	G, M, C1q	Mild	Mild	Present	Present	Present	Present	GBM	LN Class V
13 [2]	M	49	G, A	Present	Present	Present	Present	Absent	Present	Absent	MPGN (Type 3)
14 [2]	F	20	G	Absent	Absent	Present	NR	Present	Absent	GBM	MGA
15 [2]	F	47	G, A, M	Absent	Absent	Present	NR	Present	Absent	GBM	MGA
16 [2]	F	51	All negative	Absent	Absent	Present	Present	Present	Absent	Absent	MGA
17 [2]	F	30	G	Absent	Absent	Present	Present	Present	Absent	Absent	MGA
18 [2]	F	54	All negative	Present	Present	Present	Present	Present	Absent	Absent	FSGS
19 [2]	F	57	All negative	Absent	Absent	Present	Present	Present	Absent	Absent	FSGS
20 [2]	M	45	G, A, C3	Absent	Present	Present	Present	Present	Present	Absent	FSGS
21 [2]	F	42	G	Present	Absent	Present	Present	Present	Absent	Absent	FSGS + MN
22 [2]	F	69	G, A, M, C3	Absent	Absent	Present	Present	Present	Absent	Absent	MN
23 [2]	M	46	G	Absent	Absent	Present	Present	Present	Present	Absent	MN
24 [2]	M	59	M	Absent	Absent	Present	Present	Present	Absent	Absent	MN
25 [2]	F	45	G, A, C3	Absent	Absent	Present	Present	Present	Present	Absent	MN
26 [3]	M	4	All negative	NR	NR	Present	Present	Present	Absent	Absent	Focal or global sclerosis
27 [4]	F	61	M, C3	Present	Present	Present	Present	Present	Absent	Absent	LN
28 [5]	F	45	G, C3	Absent	Absent	Present	Present	Present	Absent	Absent	MN
29 [6]	F	33	M, G, C1q, C3	Mild	Mild	Present	Present	Present	Absent	Mesangium, subepithelial	Proliferative glomerulonephritis
30 [7]	F	27	M	Present	Mild	Present	Present	Present	Absent	Absent	Chronic interstitial nephritis
31 [7]	F	23	M	Absent	Absent	Present	Present	Present	Absent	Absent	MGA
32 [8]	F	14	M, C3, C1q	Absence	Absence	Absent	Presence	Present	Absent	Absent	FSGS
33 [9]	F	44	M	Absent	Absent	Present	Present	Present	Present	Mesangium, intramembrane	MGA
34 [11]	F	52	All negative	Absent	Absent	Absent	Present	Present	Present	Absent	Global and segmental sclerosis
35 [12]	F	52	All negative	NR	NR	Present	Present	Present	Absent	Absent	MGA
36 [13]	M	79	All negative	Absent	Absent	Present	Present	Present	Absent	Absent	MN
37 [14]	F	38	All negative	Absent	Present	Present	Present	Present	Present	Absent	FSGS
38 [15]	F	35	Full house pattern, faint IgG	Present	Mild	Present	Present	Present	Present	Mesangium	LN Class II
39 [16]	F	60	G, M, A, C3, PLA2r	Absent	Mild	Absent	Present	Present	Present	Subepithelial	MN
40 [17]	F	56	All negative	Absent	Absent	Present	Present	Absent	Absent	Absent	Acute tubular injury
41	F	26	G, M	Mild	Present	Present	Present	Present	Absent	Present	FSGS
42	M	47	G	Mild	Absent	Present	Present	Present	Absent	Absent	FSGS
43	F	48	A, M	None	Present	Present	Present	Present	Absent	Absent	MN
44	F	57	All negative	Absent	Mild	Mild	Present	Present	Present	Absent	MGA

Abbreviations: A, IgA; F, female; FPE, foot process effacement; FSGS, focal segmental glomerular sclerosis; G, IgG; GBM, glomerular basement membrane; IF, immunofluorescent; LM, light microscopy; LN, lupus nephritis; M, male; M (in IF staining column), IgM; MGA, mild glomerular abnormality; MN, membranous nephropathy; MPGN, membranous proliferative glomerulonephritis; NR, not reported.

## Data Availability

The data and materials reported in this work are available on reasonable request from the correspondence.

## Supplementary Materials

The following supporting information can be downloaded at: https://www.mdpi.com/article/10.3390/jcm12031088/s1, Section S1. Literature review methodology; Figure S1: Demographic characteristics of podocyte infolding cases in literature and this case series report; Figure S2: Clinical characteristics of podocyte infolding cases in literature and this case series report; Figure S3: Histological characteristics of podocyte infolding cases in literature and this case series report.

## References

[1] Sato H., Saito T., Yoshinaga K. (1992). Intramembranous fine deposit disease associated with collagen disorders: A new morphological entity?. Virchows Arch A.

[2] Joh K., Taguchi T., Shigematsu H., Kobayashi Y., Sato H., Nishi S., Katafuchi R., Nomura S., Fujigaki Y., Utsunomiya Y. (2008). Proposal of podocytic infolding glomerulopathy as a new disease entity: A review of 25 cases from nationwide research in Japan. Clin. Exp. Nephrol..

[3] Xiong S., Shuai L., Li X., Dang X., Wu X., He Q. (2020). Podocytic infolding in Schimke immuno-osseous dysplasia with novel SMARCAL1 mutations: A case report. BMC Nephrol..

[4] Liu X., Huang J., Zhang K., Niu Y., Liu Y., Cui C., Yu C. (2021). A case of Podocytic Infolding Glomerulopathy with SLE and literature review. BMC Nephrol..

[5] Matthai S.M., Mohapatra A., Mathew A.J., Roy S., Varughese S., Danda D., Tamilarasi V. (2018). Podocyte Infolding Glomerulopathy (PIG) in a Patient with Undifferentiated Connective Tissue Disease: A Case Report. Am. J. Kidney Dis..

[6] Shi J., Zheng R., Gao H., Zhao Z., Wu H., Zhang Z. (2020). Podocyte infolding glomerulopathy with undifferentiated connective tissue disease: A case report. Ultrastruct. Pathol..

[7] Zhang T., Sun W., Xue J., Chen J., Jiang Q., Mou L., Du H. (2019). Podocytic infolding glomerulopathy: Two new cases with connective tissue disease and literature review. Clin. Rheumatol..

[8] Iguchi A., Sohma A., Yamazaki H., Ito T., Saeki T., Ito Y., Imai N., Osawa Y., Narita I. (2013). A case of podocytic infolding glomerulopathy with focal segmental glomerulosclerosis. Case Rep. Nephrol. Urol..

[9] Kwon K.W., Jeong H.J., Lee J.H. (2016). Podocytic infolding glomerulopathy: A case report. Ultrastruct. Pathol..

[10] Ou J., Zhu L.J. (2019). trackViewer: A Bioconductor package for interactive and integrative visualization of multi-omics data. Nat. Methods.

[11] Ting J.A., Hung W., McRae S.A., Barbour S.J., Copland M., Riazy M. (2021). Podocyte Infolding Glomerulopathy, First Case Report from North America. Can J. Kidney Health Dis..

[12] Fang J.Y., Song A.H., Shen B., Liu Y.L. (2018). A Case of Podocytic Infolding Glomerulopathy with Primary Sjogren’s Syndrome and Hashimoto’s Thyroiditis. Chin. Med. J..

[13] Harada M., Kamijo Y., Ehara T., Shimojo H., Shigematsu H., Higuchi M. (2014). A case of podocytic infolding glomerulopathy with multiple myeloma. BMC Nephrol..

[14] Malvar A., Davila P., Ferrari M., Delgado P., Iscoff P., Lococo B., Alberton V. (2020). Podocyte infolding glomerulopathy; report of the first case in Latin America and review of the literature. Nefrologia.

[15] Manabe S., Sato M., Kataoka H., Taneda S., Mochizuki T., Nitta K. (2020). Cell invasion in glomerular basement membrane: Infolding glomerulopathy. Kidney Int..

[16] Pandit A.P., Rennke H.G., Denker B.M. (2021). Podocytic Infolding Glomerulopathy in a Patient with Phospholipase A2 Receptor-Positive Membranous Nephropathy and Review of the Literature. Nephron.

[17] Wostmann F., Muller R.U., Gobel H., Benzing T. (2019). Becker JU, Bartram MP: Case report: A peculiar glomerulopathy in a patient suffering from nephrotic syndrome. BMC Nephrol..

[18] Hinglais N., Kazatchkine N.D., Bhakdi S., Appay M.-D., Mandet C., Grossetete J., Bariety J. (1986). Immunohistochemical study of the C5d-9 complex of complement in human kidneys. Kidney Int..

[19] Subramanian B., Chun J., Perez-Gill C., Yan P., Stillman I.E., Higgs H.N., Alper S.L., Schlondorff J.S., Pollak M.R. (2020). FSGS-Causing *INF2* Mutation Impairs Cleaved *INF2* N-Fragment Functions in Podocytes. J. Am. Soc. Nephrol..

[20] Zhao Y., Zhang H., Wang H., Ye M., Jin X. (2022). Role of formin *INF2* in human diseases. Mol. Biol. Rep..

[21] Panzer L., Trube L., Klose M., Joosten B., Slotman J., Cambi A. (2016). Linder S: The formins FHOD1 and *INF2* regulate inter- and intra-structural contractility of podosomes. J. Cell Sci..

[22] Krendel M., Pruyne D. (2020). New Paradigm for Cytoskeletal Organization in Podocytes: Proteolytic Fragments of *INF2* Formin Function Independently of *INF2* Actin Regulatory Activity. J. Am. Soc. Nephrol..

