# Supplementary material for: Podocyte Infolding Glomerulopathy: A Case Series Report and Literature Review
Source: J Clin Med. 2023 Jan 30;12(3):1088. doi: 10.3390/jcm12031088 (PMC9918010; doi:10.3390/jcm12031088)
Supplement: Supplementary file 1 [file jcm-12-01088-s001.zip › jcm-2171287-supplementary.pdf]

Supplementary Materials

Section S1. Literature review methodology.

Section S2. Supplementary figures.

Figure S1. Demographic characteristics of podocyte infolding cases in literature and this case series report.

Figure S2. Clinical characteristics of podocyte infolding cases in literature and this case series report.

Figure S3. Histological characteristics of podocyte infolding cases in literature and this case series report.

Section S1. Literature review methodology

Data sources and searches

A literature search was performed for eligible studies published up to 1st July, 2022 in MEDLINE via PubMed (from 1946 through July 2022). The search term was "podocyte infolding". The search was limited to publication in English.

Study selection

Case studies on podocyte infolding (PIG) were included in this review. Two authors (Y.L.F. and W.W.) carefully and independently reviewed titles and abstracts of all returned records, and included all publications that reported one or more PIG cases after full text reviewing. Reference lists from full text reviewed articles were further manually scanned to identify any other relevant case reports. Any discrepancy was adjudicated by a third reviewer (P.Z.).

Data extraction

Two authors (Y.L.F. and W.W.) independently extracted data from eligible studies following a double-check procedure and compiled them into a shared sheet. Disagreements were resolved by the third author (P.Z.). The data extracted included sex, geographical origin, race, age, comorbidity, lab investigations at renal biopsy (including serum creatinine, proteinuria, and hematuria), treatment, response, light microscopic results of renal biopsy (including IF staining, hypercellularity, mesangial deposit, and GBM thickening), electron microscopic results of renal biopsy (including foot process effacement, microspheres, microtubules, and dense deposits), and pathological diagnosis.

Responses were categorized into four classes as follows: CR: proteinuria <0.5 g/day and serum creatinine kept normal after treatment; PR: proteinuria >0.5 g/day but deceased by ≥50% compared with the level before treatments; unchanged: proteinuria deceased by <50% compared with the level before treatments; progression: proteinuria or serum creatinine increased after treatment.

Histological results of renal biopsy were recorded according to the descriptions from the original case reports or determined according to the figures in the original publications by the author (P.Z.) who had over ten years of experience in renal pathology.

Data analysis

GraphPad Prism (version 8.0; GraphPad, MD, USA) was used for the data analysis. Descriptive data was expressed in terms of median (interquatile range) or mean ± standard deviation for continuous data, and numbers and frequency for categorical data.

Section S2. Supplementary Figures

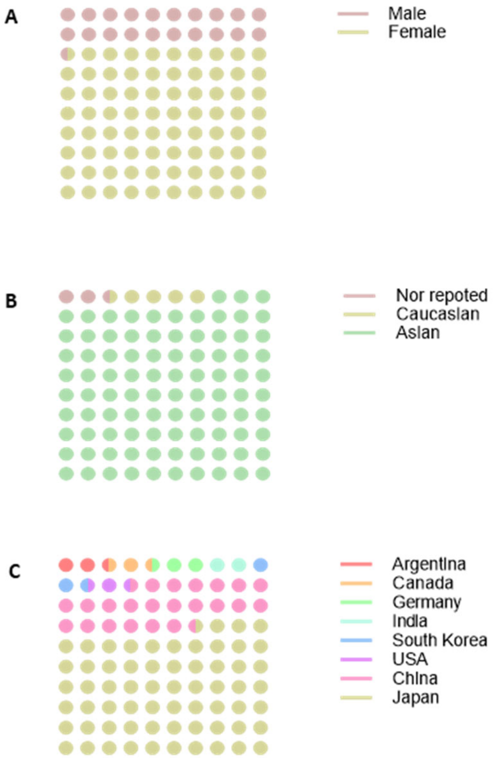

Figure S1. Demographic characteristics of podocyte infolding cases in literature and this case series report. Note: The compositions of gender (**A**), ethnics (**B**), and nationality (**C**) of podocyte infolding cases in literature and this case series report.

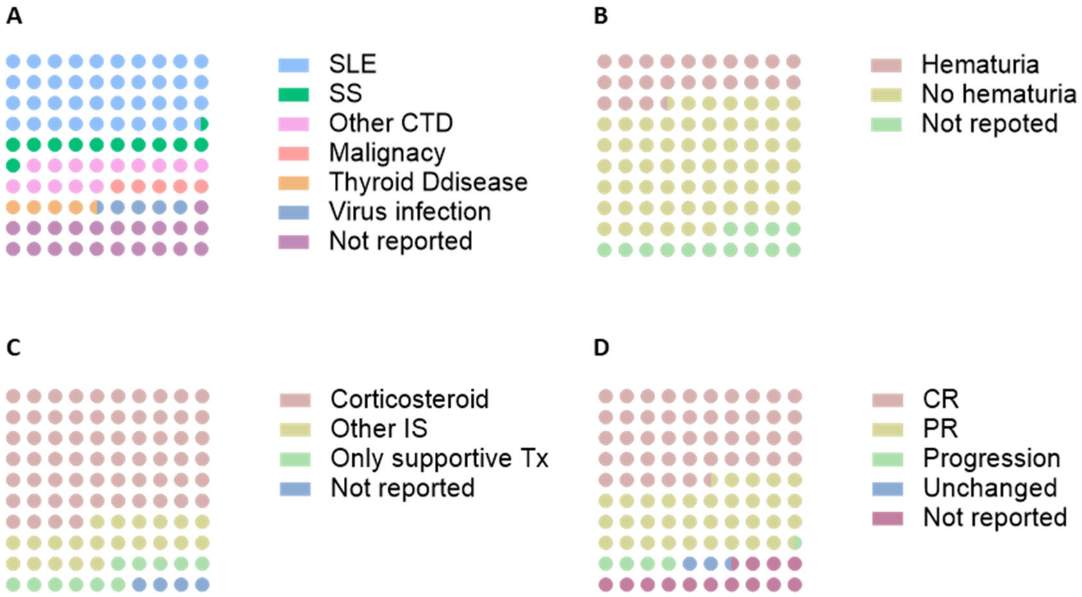

Figure S2. Clinical characteristics of podocyte infolding cases in literature and this case series report. Note: The compositions of comorbidities (**A**), appearance of hematuria at renal biopsy (**B**), therapies (**C**), and response after treatment (**D**) of podocyte infolding cases in literature and this case series report.

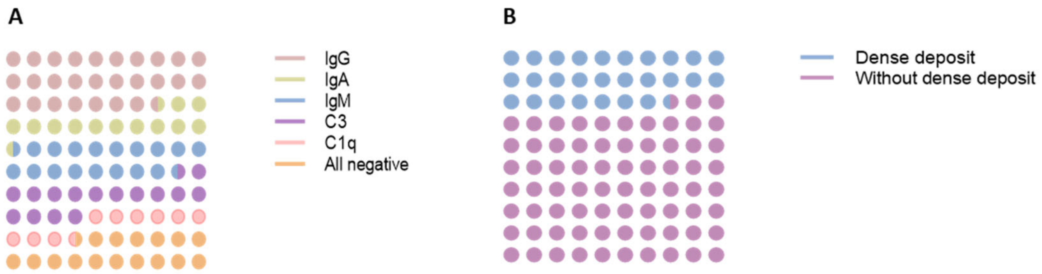

Figure S3. Histological characteristics of podocyte infolding cases in literature and this case series report. Note: The compositions of immunofluorescent staining positive proteins (**A**) and appearance of dense deposit under electronic microscopy (**B**) of podocyte infolding cases in literature and this case series report.
